# Facial Nerve Hemangioma: Radiological Findings and a Review of the Literature

**DOI:** 10.7759/cureus.105419

**Published:** 2026-03-18

**Authors:** Kawtar Khaissidi, Zaid Ennasery, Hajar Ouazzani, Ismail Chaouche, Amal Akammar, Meriem Haloua, Badreddine Alami, Moulay Youssef Alaoui Lamrani, Meryem Boubbou, Mustapha Maaroufi, Nizar El Bouardi

**Affiliations:** 1 Radiology Department, Hassan II University Hospital, Fez, MAR; 2 Mother and Child Radiology Department, Hassan II University Hospital, Fez, MAR

**Keywords:** computed tomography (ct), facial nerve hemangioma, magnetic resonance imaging (mri), radiologic findings, temporal bone

## Abstract

Facial nerve hemangioma is a rare vascular lesion of the temporal bone, most frequently arising from the geniculate ganglion of the facial nerve. Imaging plays a pivotal role in its detection and characterization, and the combination of computed tomography (CT) and magnetic resonance imaging (MRI) may allow the diagnosis based on suggestive radiologic features. We report a case of a middle-aged female with a facial nerve hemangioma diagnosed through CT and MRI, emphasizing the main imaging findings and their correlation with previously published descriptions in the literature.

## Introduction

Facial nerve hemangioma is a rare benign vascular lesion of the temporal bone, most often arising from the geniculate ganglion. MRI is central for diagnosis, while temporal bone CT is complementary by demonstrating bony changes, helpful in differentiating it from schwannoma. We report a case of a 48-year-old female with left hypoacusis and preserved facial nerve function, in whom MRI and CT findings were highly suggestive of a geniculate ganglion facial nerve hemangioma [1]. We also provide a focused review of the literature outlining the pathological basis of this entity, its key radiologic and semiologic features, as well as management considerations.

## Case presentation

A 48-year-old female with a medical history of bacterial meningitis and hypothyroidism presented with left-sided hypoacusis. Neurological examination showed no evidence of facial nerve palsy.

Brain MRI demonstrated a small nodular lesion along the intratemporal course of the left facial nerve, centered at the geniculate ganglion. The lesion appeared isointense to brain parenchyma on T1-weighted images and heterogeneously hyperintense on T2-weighted sequences with punctate hypointense foci. High-resolution sequences, including 3D fast imaging employing steady-state acquisition (FIESTA), allowed better visualization of the lesion. These imaging findings were highly suggestive of a vascular lesion arising from the geniculate ganglion.

**Figure 1:**
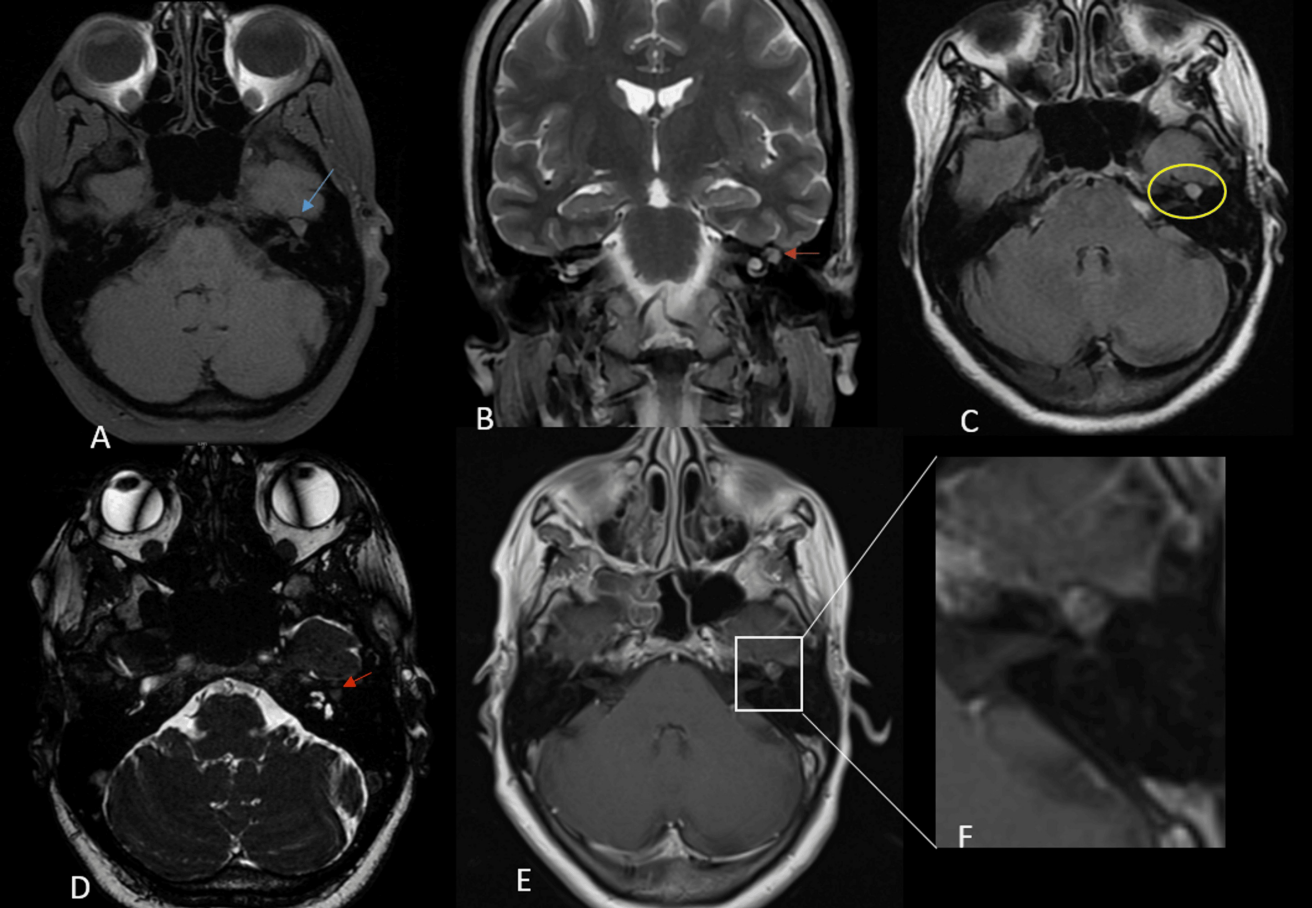
Brain MRI findings. Axial T1-weighted image (A), coronal T2-weighted image (B), axial fluid-attenuated inversion recovery (FLAIR) image (C), axial 3D fast imaging employing steady-state acquisition (FIESTA) sequence (D), and post-contrast axial T1-weighted image (E) with a zoomed-in view centered on the temporal lesion (F). MRI demonstrates a small nodular lesion along the intratemporal course of the left facial nerve, centered at the geniculate ganglion. The lesion is isointense on T1-weighted images (blue arrow), heterogeneously hyperintense on T2-weighted images with punctate hypointense foci (orange arrow), with slight hyperintensity on the 3D FIESTA sequence (red arrow). Post-gadolinium images demonstrate heterogeneous enhancement with punctate non-enhancing areas (white rectangle).

After gadolinium administration, the lesion showed intense and heterogeneous enhancement, with punctate areas of non-enhancement that correspond to the stippled hypo-intense foci (Figure 1). No significant mass effect on adjacent structures was observed.

**Figure 2:**
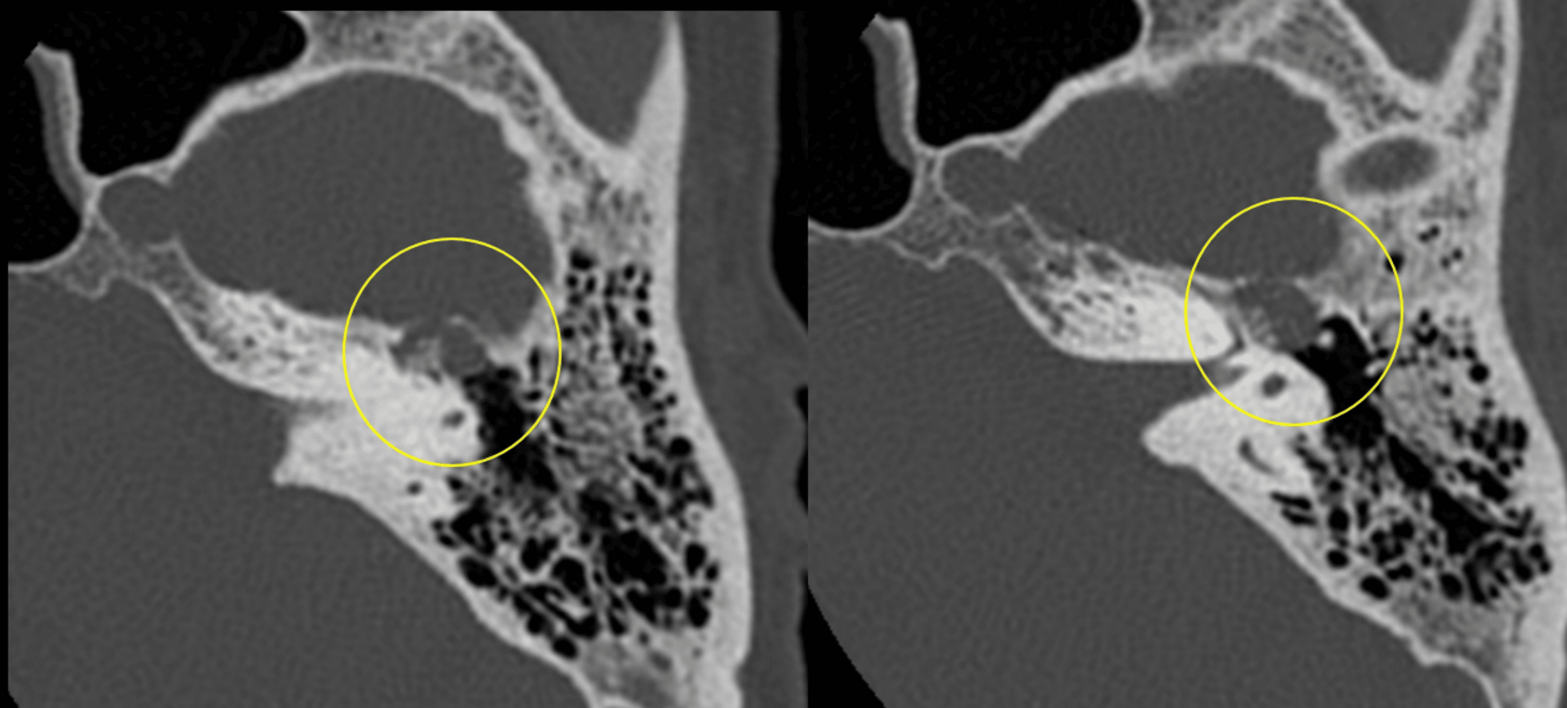
Axial thin-section CT image of the left temporal bone shows an expansile lytic lesion centered at the geniculate ganglion, containing fine punctate calcifications and delicate internal bony spicules, producing a characteristic “honeycomb” appearance (yellow circle), highly suggestive of a facial nerve hemangioma.

Subsequently, a high-resolution thin-section CT scan of the left temporal bone was performed, demonstrating an expansile lytic lesion centered in the region of the geniculate ganglion, with fine internal high-attenuation spicules consistent with intralesional calcifications. The presence of these delicate bony spicules producing a characteristic “honeycomb” pattern within the geniculate fossa is a classic and highly suggestive imaging feature of facial nerve hemangioma, and helps differentiate it from more common facial nerve tumors such as schwannoma (Figure 2).

The lesion’s location and MRI signal characteristics, including its enhancement pattern, were highly suggestive of a facial nerve hemangioma. High-resolution temporal bone CT further supported this diagnosis by demonstrating typical intralesional calcifications and delicate bony spicules within the geniculate fossa, producing a characteristic “honeycomb” appearance.

Given the preserved facial nerve function and the absence of aggressive radiological features, conservative management with MRI follow-up was recommended.

## Discussion

Facial nerve hemangiomas are rare lesions, accounting for approximately 0.7% of all intratemporal tumors [1]. They typically occur between the third and sixth decades of life, with a nearly equal sex distribution [2]. In the majority of reported cases, facial nerve hemangiomas manifest primarily with facial nerve palsy, with or without associated auditory dysfunction. Conductive hearing loss is typically related to tumor extension into the middle ear, which was not observed in our case. An extensive review of the literature has shown that only approximately 6% of hearing loss cases associated with facial nerve hemangiomas involve lesions arising from the geniculate ganglion, as in our case [3,4].

Histologically, facial nerve hemangiomas correspond to benign vascular lesions of uncertain origin, most likely arising from the perineural venous plexus, as suggested by their predilection for anatomical regions rich in this plexus, particularly the geniculate fossa [1].

In the literature, some authors classify facial nerve hemangiomas as vascular malformations rather than tumors [5]. Facial nerve hemangiomas may involve different segments of the facial nerve, most commonly the geniculate ganglion, followed by the internal auditory canal, with rare involvement of the posterior genu or mastoid segment. Reported tumor sizes range from a few millimeters to 2 cm, with the majority measuring less than 1 cm [5,3].

On MRI, facial nerve hemangiomas typically appear iso- to hypointense relative to the brain parenchyma on T1-weighted images and hyperintense on T2-weighted sequences. Small punctate hypointense foci, point-like low-signal areas visible on both T1- and T2-weighted images, may be present and are thought to correspond to calcified or ossified components within the lesion.

High-resolution, heavily T2-weighted three-dimensional sequences, such as FIESTA, significantly improve the detection of small lesions and allow a more accurate assessment of their extent compared with conventional two-dimensional sequences.

After administration of gadolinium-based contrast material, facial nerve hemangiomas consistently demonstrate marked enhancement, which is most often heterogeneous, although homogeneous enhancement has also been reported [6]. The heterogeneous signal reflects the vascular nature of the lesion, related to slow-flow venous channels and occasional thrombosis.

A geniculate ganglion-centered lesion showing intense contrast enhancement should strongly suggest a facial nerve hemangioma, particularly in the differential diagnosis with facial nerve schwannoma, Bell’s palsy, or malignant perineural spread. In contrast to schwannomas, which typically present as smooth fusiform nerve enlargement, hemangiomas are often small yet demonstrate disproportionately intense enhancement, making MRI essential for diagnosis [7].

High-resolution temporal bone CT plays a key complementary role in the evaluation of suspected facial nerve hemangioma. Typical CT findings include an irregular, mildly expansile lytic lesion centered on the geniculate fossa, often containing fine internal high-attenuation punctate calcifications and or delicate osseous spicules within the tumor matrix. When present, this intralesional ossified matrix produces the characteristic “honeycomb” appearance, which is considered virtually pathognomonic for facial nerve hemangioma and helps differentiate it from facial nerve schwannoma, which more commonly causes smooth expansion of the facial nerve canal. However, this classic honeycomb pattern is reported in only about 50% of cases [1]. Recent radiological literature emphasizes that imaging findings, combined with clinical data, can be sufficient to suggest the diagnosis and guide management. In patients with preserved facial nerve function, as in our case, conservative management with serial imaging follow-up is increasingly advocated [8,9].

## Conclusions

Facial nerve hemangioma should be considered in the differential diagnosis of enhancing geniculate ganglion lesions. MRI alone can provide sufficient diagnostic clues through lesion location, signal characteristics, and enhancement pattern. However, combining MRI with high-resolution temporal bone CT can further improve diagnostic confidence, as CT may demonstrate characteristic osseous changes that are highly suggestive of this entity. In asymptomatic patients or those with preserved facial nerve function, MRI-based surveillance represents a safe and effective management strategy.
